# Differences in Prostate Cancer Incidence and Mortality in Lower Saxony (Germany) and Groningen Province (Netherlands): Potential Impact of Prostate-Specific Antigen Testing

**DOI:** 10.3389/fonc.2021.681006

**Published:** 2021-05-28

**Authors:** Sanny Kappen, Geertruida H. de Bock, Eunice Sirri, Claudia Vohmann, Joachim Kieschke, Alexander Winter

**Affiliations:** ^1^ Division of Epidemiology and Biometry, Department of Health Services Research, School of Medicine and Health Sciences, Carl von Ossietzky University Oldenburg, Oldenburg, Germany; ^2^ Department of Epidemiology, University Medical Center Groningen, University of Groningen, Groningen, Netherlands; ^3^ Lower Saxony Epidemiological Cancer Registry, Oldenburg, Germany; ^4^ University Hospital for Urology, Klinikum Oldenburg, Department of Human Medicine, School of Medicine and Health Sciences, Carl von Ossietzky University Oldenburg, Oldenburg, Germany

**Keywords:** prostatic neoplasms, incidence, mortality, epidemiology, early detection of cancer, prostate-specific antigen, Germany, Netherlands

## Abstract

**Background:**

Prostate cancer (PCa) is the most frequent cancer among men in Europe. Differences in PCa incidence around the world can be partly explained by variations in recommendations for prostate-specific antigen (PSA), particularly for early detection. For example, the PSA testing policy is more conservative in the Netherlands than in Germany. To better understand the relationship between PSA testing recommendations and PCa incidence, stage distribution, and mortality, we compared these variables over time between Lower Saxony in northwestern Germany and the neighboring province of Groningen in the Netherlands.

**Methods:**

Population data, tumor stage- and age group-specific PCa incidence (ICD-10 C61) and mortality rates for Lower Saxony and Groningen were obtained from the Lower Saxony Epidemiological Cancer Registry, the Netherlands Comprehensive Cancer Organization, and Statistics Netherlands for 2003–2012. Incidence and mortality rates per 100,000 person-years were age-standardized (ASR, old European standard). Trends in age-standardized incidence rates (ASIR) and mortality rates (ASMR) for specific age groups were assessed using joinpoint regression.

**Results:**

The mean annual PCa ASIR between 2003 and 2012 was on average 19.9% higher in Lower Saxony than in Groningen (120.5 vs. 100.5 per 100,000), while the mean annual ASMR was on average 24.3% lower in Lower Saxony than in Groningen (21.5 vs. 28.4 per 100,000). Between 2003 and 2012, the average annual percentage change (AAPC) in PCa incidence rates did not change significantly in either Lower Saxony (−1.8%, 95% CI −3.5, 0.0) or Groningen (0.2%, 95% CI −5.0, 5.7). In contrast, the AAPC in mortality rate decreased significantly during the same time period in Lower Saxony (−2.5%, 95% CI −3.0, −2.0) but not in Groningen (0.1%, 95% CI −2.4, 2.6).

**Conclusions:**

Higher PCa incidence and lower PCa-related mortality was detected in Lower Saxony than in Groningen. Although recommendations on PSA testing may play a role, the assessed data could not offer obvious explanations to the observed differences. Therefore, further investigations including data on the actual use of PSA testing, other influences (e.g., dietary and ethnic factors), and better data quality are needed to explain differences between the regions.

## Introduction

GLOBOCAN 2018 reported that prostate cancer (PCa) is the second most common cancer in men and the fifth leading cause of death worldwide. In that year, an estimated 1,276,106 new PCa cases were reported worldwide, with higher prevalence in developed countries (1, 2). PCa incidence rates varied more than 17-fold between nations. The highest age-standardized rates (ASR, world standard) were found in Australia/New Zealand and Northern Europe (86 per 100,000), Western Europe (76 per 100,000), and North America (74 per 100,000) and the lowest rates were observed in South-Eastern Asia (13 per 100,000) and South-Central Asia (5 per 100,000) (1). One explanation for the global variations in PCa incidence is differences in the use of serum prostate-specific antigen (PSA) testing, which detects a glycoprotein that is normally expressed by prostate tissue and is elevated in most PCa types (1, 3–5).

Political decisions, results of clinical studies, and guidelines on PSA testing each play an important role in setting national policies for the early detection and treatment of PCa (1, 3, 6–8). The results of the European Randomized Study of Screening for Prostate Cancer (ERSPC) showed that PCa mortality rates can be decreased by PSA testing (9–12), whereas earlier results from the Prostate, Lung, Colorectal and Ovarian (PLCO) trial showed that screening was not associated with reduced PCa mortality rates (13–15). However, later results indicated that contamination by opportunistic screening substantially limited the ability of the PLCO trial to identify a statistically significant screening benefit (13, 14, 16–18). Taking these results into account, the US Preventive Task Force reported that PSA screening can potentially reduce deaths from PCa in men aged 55–69 years. In determining whether this service is medically useful in men of these ages, patients and physicians were advised to balance the benefits and harms of screening while considering factors such as family history, race/ethnicity, and comorbidities (19). At the same time, PSA-based screening in men aged ≥70 years was not recommended (19). In the Cluster Randomized Trial of PSA Testing for Prostate Cancer (CAP) study in Great Britain, 419,582 men were randomized to a single PSA screening intervention versus standard practice of no screening, and the groups showed no significant difference in PCa mortality after a median follow-up of 10 years, although the detection of low-risk PCa cases was higher in the PSA screening group (20, 21). A recent systematic review concluded that, at best, PCa screening leads to a small reduction in disease-specific mortality over 10 years but has no effect on overall mortality (22). Notably, the results of the aforementioned studies, as well as guidelines and recommendations based on them, have influenced the use of PSA testing by men and their physicians (6, 8).

The national policy on PSA testing, as reflected in national guidelines, is more conservative in the Netherlands than in Germany. For example, the German S3 guideline on PCa has long recommended proactively informing men of PSA testing as an individual screening method, while the Dutch Urological Association guidelines (Nederlandse Vereniging voor Urologie) recommend against actively offering PSA testing to men without clinical symptoms of PCa (23–26). In Germany, the first guideline on PCa was published by the German Urological Association (Deutsche Gesellschaft für Urologie e.V.) in 2009 and an update was published in 2011, and in the Netherlands the first guideline on PCa was published in 2007 (with the next update in 2014) (24, 26–28). During the study period from 2003 to 2012, the national recommendations on PCa early detection did not change noticeably in both countries.

The aim of the present study was to describe and compare PCa incidence, stage distribution, and mortality rates to better understand the possible impact of national PSA testing strategies on these factors. To reduce the influence of potential confounders, we selected two demographically comparable and geographically neighboring regions for comparison: Lower Saxony in Germany and the province of Groningen in the Netherlands.

## Methods

### Data Source

All datasets encompassed the years 2003 to 2012. For Groningen province, data on population and mortality were obtained from Statistics Netherlands (Centraal Bureau voor de Statistiek), and data on incidence and tumor sizes (tumor [T] stages T1, T2, T3, T4, TX [unknown]) for different age groups were obtained from the Netherlands Comprehensive Cancer Organization (Integraal Kankercentrum Nederland). For Lower Saxony, data on population, incidence, tumor sizes, and mortality were provided by the Lower Saxony Epidemiological Cancer Registry (Epidemiologisches Krebsregister Niedersachsen). PCa cases were defined using the International Classification of Diseases, Version 10 of the World Health Organization (ICD-10 C61). Cases registered based on the death certificate alone were excluded from the incidence computations due to high percentage of missing tumor characteristics like the Tumor–Node–Metastasis (TNM) Classification of Malignant Tumors and the fact that in these cases the diagnosis date is often unclear. As a basic indicator of cancer registry data quality, the completeness of cases for the two registries was >90% for all years. In contrast to the Dutch, the Lower Saxony registry was a comparatively young registry in 2003. The proportion of death certificate only cases was 14.7% in 2003 and declined to 5.5% in 2012. T stage data were coded according to the TNM Classification valid at the moment of incidence. For incidences from 2003 to 2009 this was the TNM classification 6^th^ edition, and for incidences from 2010 to 2012 the TNM classification 7^th^ edition. N and M stage data were not considered due to the high percentage of missing information.

### Statistical Analysis

Population, incidence, and mortality data were used to calculate ASR for PCa incidence (ASIR) and mortality (ASMR) using the old European standard population. The following age groups (in years) were used for standardization: 0–49, 50–59, 60–64, 65–69, 70–74, 75–79, 80–84, and ≥85. Trends for yearly incidence and mortality rates are reported as ASR per 100,000, with all age groups considered. Annual age-specific incidence rates (in 10-year age groups, ASR calculated by using 5-year age groups) were computed. The distribution of incidence cases by T stage was described. Here, TX stages were excluded in the main results but are reported in the **Supplementary Data**. Trends in overall age-standardized and age group-specific incidence and mortality rates for men aged ≥50 years were assessed using Joinpoint Regression Program version 4.8.0.1 (27). A log-linear regression model was employed to compute average annual percentage changes (AAPC), annual percentage changes (APC), and 95% confidence intervals (CI). The t test was used to determine whether AAPC and APC were significantly different from zero at the alpha = 0.05 level. Data were analyzed using IBM SPSS version 25 (29).

## Results

### PCa Incidence Rates in Lower Saxony and Groningen From 2003 to 2012

#### Age-Standardized PCa Incidence Rates

**Table 1** shows the PCa ASIR rates for Lower Saxony and Groningen from 2003 to 2012. In Lower Saxony, the ASIR per 100,000 ranged from 110.3 in 2012 to 131.0 in 2003, while the rates in Groningen ranged from 85.3 in 2010 to 115.3 in 2005. Thus, the average ASIR was 19.9% higher in Lower Saxony than in Groningen (120.5 vs. 100.5 per 100,000).

**Table 1:** Age-standardized prostate cancer incidence rates in men of all ages in Lower Saxony and Groningen province from 2003 to 2012.

Year	Lower Saxony			Groningen Province		
	Cases	ASR	95% CI	Cases	ASR	95% CI
2003	6687	131.0	127.9, 134.1	307	99.7	88.5, 110.9
2004	6448	123.6	120.6, 126.6	339	108.7	97.1, 120.3
2005	6385	119.5	116.6, 122.4	363	115.3	103.4, 127.2
2006	6400	118.1	115.2, 121.0	349	108.2	96.8, 119.6
2007	6789	123.2	120.3, 126.1	336	102.2	91.3, 113.1
2008	6686	119.6	116.7, 122.5	316	93.1	82.8, 103.4
2009	6988	122.4	119.5, 125.3	337	91.8	82.0, 101.6
2010	6981	120.3	117.5, 123.1	307	85.3	75.8, 94.8
2011	6875	116.8	114.0, 119.6	387	105.3	94.8, 115.8
2012	6557	110.3	107.6, 113.0	361	95.4	85.6, 105.2
Average	6680	120.5	117.6, 123.4	340	100.5	89.8, 111.2

ASR, age-standardized rate (old European) per 100,000; CI, confidence interval.

#### Trend Analysis of the Overall Age-Standardized PCa Incidence Rates

**Figure 1:**
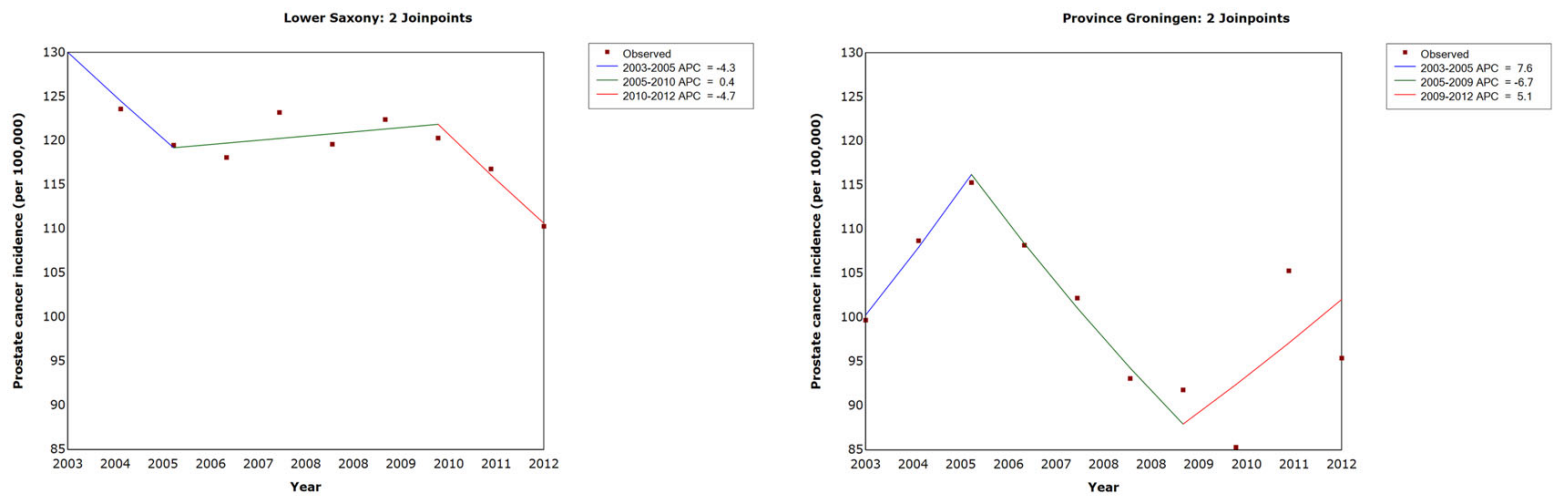
Trends in age-standardized prostate cancer incidence rates in men of all ages in Lower Saxony and Groningen province from 2003 to 2012. APC, annual percentage change in age-standardized rate (old European) per 100,000.

**Figure 1** and **Table 2** show the results of the trend analysis. The AAPC and APC for Lower Saxony and Groningen did not vary significantly between 2003 and 2012.

**Table 2:** Age-standardized prostate cancer incidence rates and trends by age group in Lower Saxony and Groningen province from 2003 to 2012.

Region	Age group(years)	Incidence rate^1^	Joinpoint analysis
			AAPC (95% CI)
Lower Saxony	All	120.5	−1.8 (−3.5, 0.0)
	50–59	127.1	−0.8 (−4.0, 2.5)
	60–69	538.6	−0.8 (−1.4, −0.2)*
	70–79	843.3	−2.5 (−4.1, −0.9)*
	≥80	563.6	−2.9 (−5.2, −0.5)*
Groningen province	All	100.5	0.2 (−5.0, 5.7)
	50–59	83.9	3.8 (−1.4, 9.2)
	60–69	412.2	−1.4 (−2.9, 0.1)
	70–79	750.3	0.4 (−5.1, 6.3)
	≥80	667.9	−2.8 (−4.0, −1.7)*

AAPC, average annual percentage change; CI, confidence interval. ^1^Average annual age-standardized (old European) incidence rate per 100,000. *Significantly different from zero (p ≤ 0.05).

#### Age Group-Specific PCa Incidence Rates and Trends

**Figure 2:**
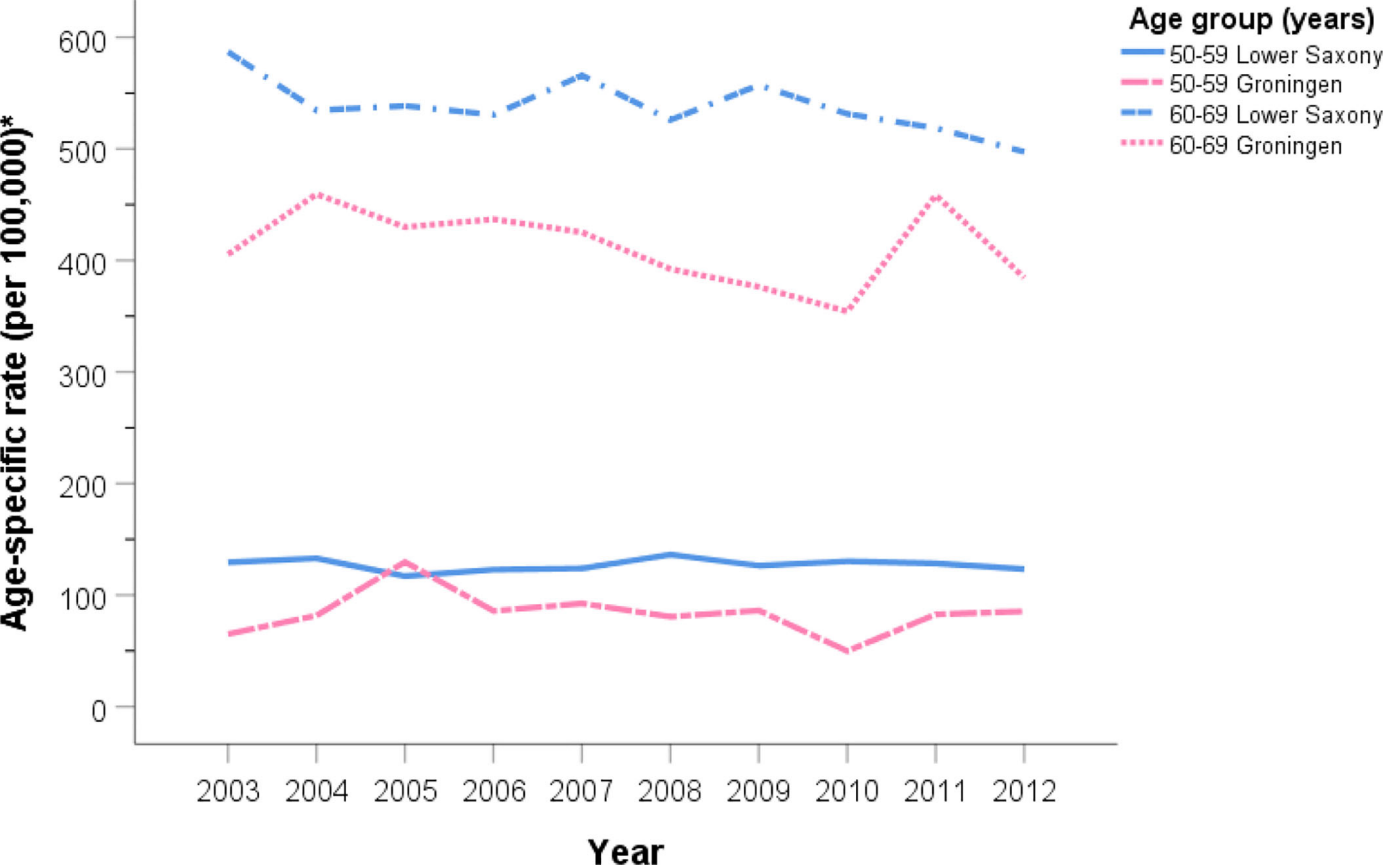
Age group-specific prostate cancer incidence rates for men aged 50–59 and 60–69 years in Lower Saxony and Groningen province from 2003 to 2012. *Age-standardized rate (old European) per 100,000.

**Figure 3:**
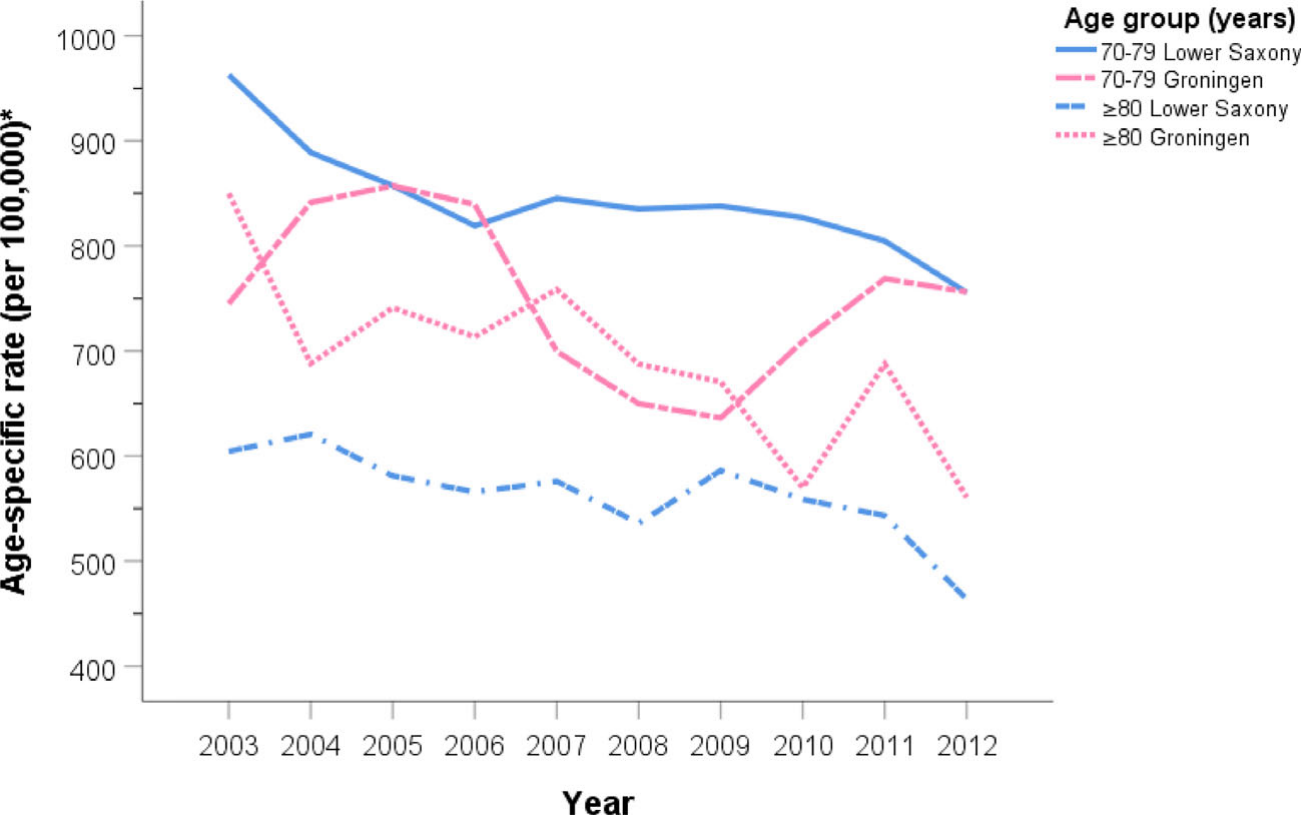
Age group-specific prostate cancer incidence rates for men aged 70–79 and ≥80 years in Lower Saxony and Groningen province from 2003 to 2012. *Age-standardized rate (old European) per 100,000.

**Figures 2** and **3** show the PCa ASIR rates in Lower Saxony and Groningen between 2003 and 2012 for each age group. With the exception of the ≥80 age group, the age group-specific rates were higher in Lower Saxony than in Groningen. For the 50–59 age group, incidence rates were constant in both regions at 100–150 per 100,000 in Lower Saxony and 50–100 per 100,000 in Groningen, except in 2005. For the 60–69 age group, rates were about 550 per 100,000 in Lower Saxony and about 400 per 100,000 in Groningen for all years. Overall, the rates for the 70–79 age group showed more fluctuation than the younger age groups, and the rates were higher in Lower Saxony than in Groningen. The ≥80 age group showed a decline in incidence rates in both regions, and this was the only age group for which the rates were higher in Groningen than in Lower Saxony.

#### Trend Analysis of the Age Group-Specific Age-Standardized PCa Incidence Rates

For men in Lower Saxony, the AAPC between 2003 and 2012 decreased significantly for the 60–69 age group (−0.8%, 95% CI −1.4, −0.2, p<0.05), the 70–79 age group (−2.5%, 95% CI −4.1, −0.9, p<0.05), and the ≥80 age group (−2.9%, 95% CI −5.2, −0.5, p<0.05). Over the same time period in Groningen, the AAPC decreased significantly only for men aged ≥80 years (−2.8%, 95% CI −4.0, −1.7, p<0.05) (**Table 2**).

#### Tumor Stage-Specific PCa Cases and Trends in Lower Saxony and Groningen

**Figure 4:**
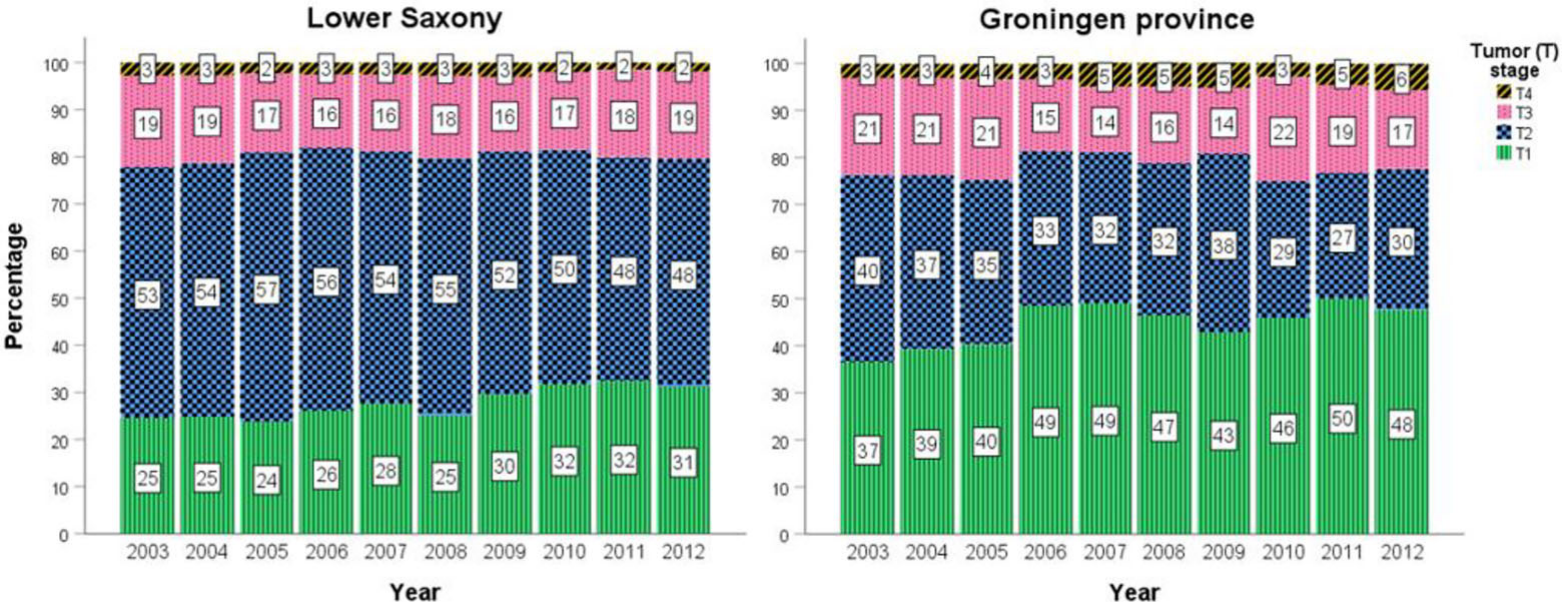
Percentage prostate cancer cases stratified by T1–T4 stage in men aged ≥50 years in Lower Saxony and Groningen province from 2003 to 2012.

**Figure 4** shows the percentage of PCa cases at T1–T4 stage for men aged ≥50 years between 2003 and 2012 for Lower Saxony and Groningen. Because the percentage of TX cases in Lower Saxony was considerably higher than in Groningen during the entire study period (up to 29.6% vs. 1.4% in 2003, 15.8% vs. 1.4% in 2012), TX cases were excluded from **Figure 4** but are included in **Supplemental Figure S1**.

In general, the percentage of T1 cases was higher in Groningen than in Lower Saxony between 2003 and 2012, while the reverse trend was observed for T2 cases. For example, T1 represented almost half of all cases in 2012 in Groningen (47.6%, n=168) compared with 31.2% (n=1700) in Lower Saxony. Conversely, the percentage of T2 cases in 2012 was lower in Groningen (30.0%, n=106) than in Lower Saxony (48.3%, n=2634). Overall, the percentages of advanced stage PCa (T3 and T4) were comparable between the two regions. Small fluctuations in the percentage of all stages, especially T1 and T2, were observed over the 10-year time frame (**Figure 4**).

### PCa Mortality Rates in Lower Saxony and Groningen From 2003 to 2012

#### Age-Standardized PCa Mortality Rates

In Lower Saxony, the PCa ASMR for men of all ages showed a steady decline from 25.3 per 100,000 in 2003 to 20.1 per 100,000 in 2012 (**Table 3**). In Groningen, the ASMR rates were more heterogenous over the 10-year span, ranging from 22.0 per 100,000 in 2004 to 35.9 per 100,000 in 2006. On average, the ASMR was 24.3% lower in Lower Saxony than in Groningen (21.5 vs. 28.4 per 100,000).

**Table 3:** Age-standardized prostate cancer mortality rates in men of all ages in Lower Saxony and Groningen province from 2003 to 2012.

Year	Lower Saxony			Groningen Province		
	Deaths	ASR	95% CI	Deaths	ASR	95% CI
2003	1234	25.3	23.9, 26.7	104	33.8	29.1, 38.5
2004	1191	23.4	21.1, 24.7	71	22.0	18.9, 25.1
2005	1226	23.3	22.0, 24.6	90	27.2	23.3, 31.1
2006	1218	22.1	20.9, 23.3	118	35.9	30.4, 41.4
2007	1131	19.8	18.6, 21.0	83	24.7	20.8, 28.6
2008	1230	20.8	19.6, 22.0	106	29.7	24.7, 34.7
2009	1230	20.0	18.9, 21.1	92	25.8	20.8, 30.8
2010	1305	20.5	19.4, 21.6	101	27.7	22.2, 33.2
2011	1327	20.1	19.0, 21.2	100	26.3	20.9, 31.7
2012	1370	20.1	19.0, 21.2	119	31.2	22.1, 40.3
Average	1246	21.5	20.3, 22.7	984	28.4	23.6, 33.2

ASR, age-standardized rate (old European) per 100,000; CI, confidence interval.

#### Trend Analysis of the Age-Standardized PCa Mortality Rates

**Figure 5:**
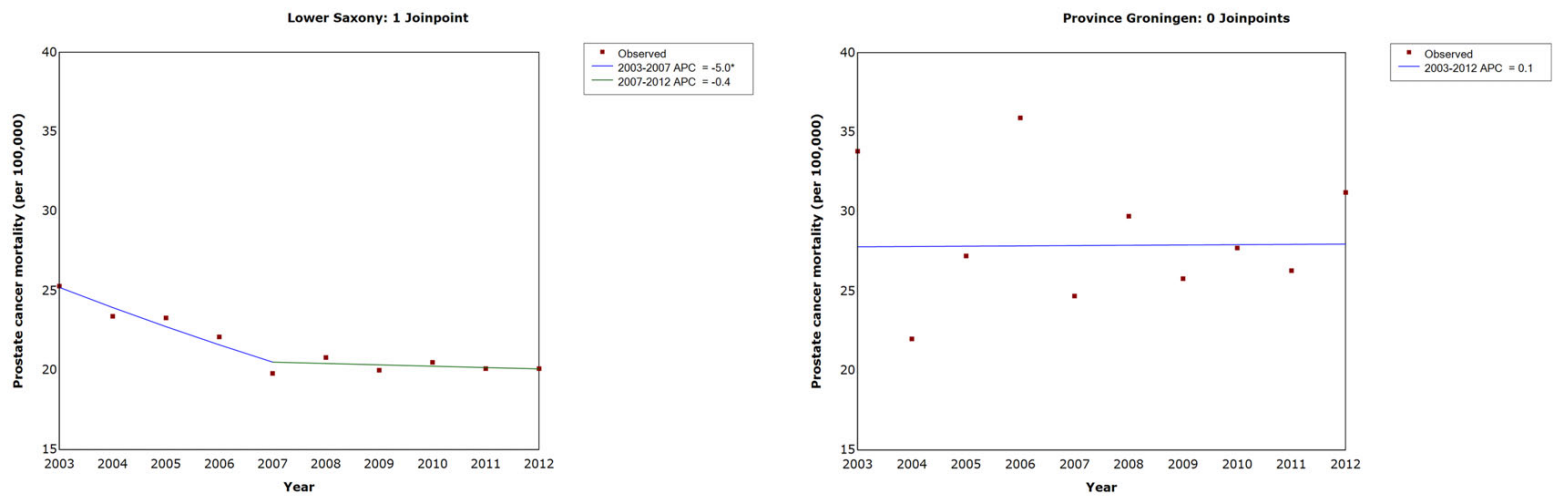
Trends in age-standardized prostate cancer mortality rates in men of all ages in Lower Saxony and Groningen province from 2003 to 2012. APC, annual percentage change in age-standardized rate (old European) per 100,000. * Significantly different from zero (p ≤ 0.05).

The results of the trend analysis for PCa mortality are shown in **Table 4** and **Figure 5**. In Lower Saxony, the AAPC decreased significantly between 2003 and 2012 (−2.5%, 95% CI −3.0, −2.0, p<0.05), while the AAPC in Groningen showed no significant change during the same period (0.1%, 95% CI −2.4, 2.6, p=1.0). In Lower Saxony, the APC in PCa mortality decreased significantly between 2003 and 2007 (−5.0%, 95% CI −6.1, −3.9) and showed a small additional decrease between 2007 and 2012 (−0.4%, 95% CI −1.2, 0.4). In Groningen, the APC in mortality rates showed small fluctuations between 2003 and 2012, but no clear trend or joinpoints were observed.

**Table 4:** Overall age-standardized prostate cancer mortality rates and trends for men of all ages in Lower Saxony and Groningen province from 2003 to 2012.

Region	Age group	Mortality rate^1^	Joinpoint analysis
			AAPC (95% CI)
Lower Saxony	All^2^	21.5	−2.5 (−3.0, −2.0)*
Groningen province	All^2^	28.4	0.1 (−2.4, 2.6)

AAPC, average annual percentage change; CI, confidence interval. ^1^Average annual age-standardized (old European) incidence rate per 100,000. ^2^Includes men in all age groups. *Significantly different from zero (p ≤ 0.05).

## Discussion

To the best of our knowledge, this is the first study to perform a detailed comparison of PCa incidence and mortality rates, T stage distribution, and time trends in two neighboring regions that follow different recommendations for PSA testing. We found that PCa ASIR were higher, while ASMR were lower, in Lower Saxony than in Groningen between 2003 and 2012. In addition, while the percentages of advanced stage PCa (T3 and T4) were comparable between the two regions, T1 cases were less frequent and T2 cases were more frequent in Lower Saxony than in Groningen during the same period. Finally, we observed that ASMR decreased in Lower Saxony but did not change significantly in Groningen from 2003 to 2012.

An analysis of PCa incidence data from the International Agency for Research on Cancer (IARC) showed increases in PCa incidence in many other European countries like Denmark, Croatia and Lithuania since the mid-1990s (30). This contrasts with the lack of significant changes in overall incidence rates between 2003 and 2012 in our trend analyses of Lower Saxony and Groningen province. The overall PSA testing rate in men aged ≥45 years in the Netherlands increased nearly 4-fold between 2002 and 2011 from 15.5 to 54.3 per 1000 person-years, despite recommendations from several sources (e.g., European and Dutch guidelines) advising a more conservative use of PSA testing (31). If PSA testing and PCa incidence are associated, it would be logical to observe that PCa incidence rates would increase in parallel with PSA testing rates. Indeed, this conclusion is supported by an analysis of PCa incidence and mortality trends in 36 countries, including the Netherlands (1, 3, 32). However, in the present study, we detected no long-term change in PCa incidence in Groningen. Several explanations for these seemingly contradictory findings are possible; for example, the rate of PSA testing in Groningen may not be comparable to that in the rest of the country; the influence of guidelines for PSA testing on PCa incidence may be less robust in that region; or other factors, such as variation in data quality and registration procedures, may have contributed. Of interest, the IARC analysis mentioned before also showed stable incidence rates in the Netherlands between 1999–2008 (30).

The higher PCa incidence rates in Lower Saxony than in Groningen identified here could be partly explained by the more intensive PSA testing recommendations in the German S3 guidelines compared with the Dutch guidelines (24, 26). This possibility is supported by the fact that all urologists surveyed in Lower Saxony claimed to have detailed knowledge of the S3 guidelines (33). The results of a survey conducted in the north-east of the Netherlands also support this possibility by stating that clinical PSA testing in primary care generally seems to be consistent with the relevant guideline for Dutch GPs that is restrictive to PSA testing (34, 35). The survey conducted in Lower Saxony, however, also included general practitioners. They disconfirmed this possibility by stating to know the recommendations of the German College of General Practice and Family Medicine (Deutsche Gesellschaft für Allgemein- und Familienmedizin) best, which also recommends against the PSA test as an individual early detection method (33, 36).

In an analysis among 43 populations worldwide, trends in PCa incidence rates showed five distinct patterns, ranging from generally monotonic increases to peaks in rates followed by declines that coincided to some extent with changes in the prevalence of PSA testing (37). In that study, ASIR was highest for men aged ≥75 years in most of the high-income populations, reflecting a decline in PSA testing use in older age groups and the diagnosis of PCa in younger age groups. Our results are consistent with this observation, as the highest annual incidence rates were detected in the 70–79-year-old groups. Analyzing age-specific PCa trends may help to identify high-risk populations and indicate associations related to the use of and recommendations for PSA testing.

Several studies support an association between widespread use of PSA testing and a shift towards earlier PCa diagnosis or localized tumor stage. A comparison of PCa incidence and mortality rates in Germany and the United States showed a significant increase in the proportion of localized disease from 51.9% (1998–2000) to 69.6% (2007–2010) in Germany, while in the United States only a small increase of about 2% was seen between 1998–2000 and 2007–2010 (5). In contrast to Germany, the United States already saw a large proportion of localized cases (80.5%) in 1998–2000. Regarding the trend in Germany, a comparable shift from capsule-exceeding to capsule-limited tumors was shown in an analysis of data from the Munich Cancer Registry, especially in the 1990s (5, 38), and similar changes in stage distribution have been shown for other European countries (39). In the United States, both the incidence of early-stage PCa and rates of PSA testing have declined since publication of the 2012 US Preventive Services Task Force recommendation to omit PSA screening from routine primary care (40). Logically, countries with more intensive PSA testing due to recommendations, such as Germany, would be expected to have a higher proportion of early-stage PCa than countries with comparatively restrictive PSA testing recommendations, such as the Netherlands. However, it is difficult to make conclusions based on T1 stage data because that is not necessarily the earliest/smallest T stage. In the present study, the percentages of T1 and T2 cases were higher and lower, respectively, in Groningen than in Lower Saxony. These differences might be due to differences in the data acquisition procedures. For example, the data for Lower Saxony included pathological and clinical reports, while in Groningen data included only clinical information. Since pathological reports often do not contain T stage information at the date of diagnosis, this may have influenced the observed proportions of T1 and T2 cases and also led to the higher proportion of TX cases in Lower Saxony than in Groningen. Furthermore, an evaluation of the association between bioptic and pathological Gleason score in a series of patients undergoing prostate needle biopsy and subsequent radical prostatectomy reported, that almost 40% of patients received an upstaging on the pathological specimen compared to the bioptic one (41). This might have been the case in this study and could partly explain the observed differences in the two regions, even as possible existing differences in preferred therapies between the regions such as the proportion of radical prostatectomies among all PCa. The proportions of T3 and T4 cases were comparable between the two regions. Additional variation in data quality, registration procedures, the proportions of clinical and pathological reporting of information in the cancer registries, and/or the reporting date (preoperative vs postoperative) could have influenced the results.

Concerning death rates for PCa, the declining mortality rates in Lower Saxony found in our study are comparable to trends in PCa mortality in Northern America, Oceania, and Northern and Western Europe (32). An analysis of trends in PCa mortality in 53 countries since 1985 found a decreasing trend in PCa mortality that was comparable for Germany and the Netherlands (30). In our study, Lower Saxony showed a significant decrease in PCa mortality between 2003 and 2012, while rates in Groningen stayed virtually the same. In the IARC analysis, the absolute mortality rate was higher in the Netherlands than in Germany. This is comparable to our results, which showed higher absolute mortality rates for Groningen than for Lower Saxony. An analysis in Norway between 2009 and 2014 showed an overreporting percentage of PCa deaths of 33% in patients registered as dead from PCa (42). Overreporting of PCa deaths may also affect PCa mortality statistics in other countries like Germany and/or the Netherlands.

There are multiple potential reasons for the differences in PCa incidence and mortality rates between the two regions in Germany and the Netherlands studied here. For example, the differences may be linked to early detection and/or improved treatment, although the contribution of PSA testing is controversial (9–15, 43, 44). Beyond the influence of recommendations for and use of PSA testing, other confounders may be relevant and affect PCa incidence and mortality rates, trends, and stage distribution. For example, differences in the ethnic composition and dietary habits of the populations must be studied (2). Furthermore, regional variations in PCa incidence can be found within both countries. The population of Groningen is less than that of Lower Saxony, and more fluctuation in incidence (wider confidence intervals) was observed in Groningen. In 2015–2016, Lower Saxony had the highest age-standardized PCa incidence among states in Germany, while Groningen had the third lowest rate among provinces in the Netherlands (45, 46). Such factors could contribute to an overestimation of differences in the results between these regions. Our study thus provides an impetus for further analysis of potential factors that may explain the differences in incidence and mortality rates between the two regions.

Apart from the potential reduction in disease-specific mortality, PSA testing may lead to overdiagnosis and overtreatment (22). The results of this study add important information to our understanding of the influence of PSA testing on PCa incidence and mortality. Strengths of this study were that to our knowledge, it is the first study to compare population-based registry data from two demographically comparable and geographically neighboring regions that differ in recommendations for PCa early detection. The results could be refined further if actual PSA test use in both regions were available. However, PSA testing is not provided as a service by the German statutory health insurance, and these data are therefore not available for Lower Saxony or other regions in Germany. In other countries, these data are often based on secondary data (47, 48). As study limitations, the age of the data and the data quality have to be mentioned. Due to the time period examined, no direct conclusions can be drawn regarding the impact of changes in recommendations on PSA testing after 2012 (e.g., US Preventive Services Task Force recommendations). Because of poor TNM data quality, incidences by Union for International Cancer Control (UICC) stage could not be computed. Also, the high proportion of cases with unknown T stage in Lower Saxony prevents drawing any meaningful conclusions from the comparison of stage distribution. Consistent registration procedures between cancer registries would be desirable to better compare data between countries. A follow-up study covering a longer time span with more recent data and better data quality regarding for example more complete information on TNM classification and specific information on PSA testing are needed to explain the differences in PCa incidence, mortality, stage distribution, and trends between Lower Saxony and Groningen province.

## Conclusion

We detected higher PCa incidence and lower mortality rate in Lower Saxony than in Groningen. Although recommendations on PSA testing may play a role, the assessed data could not offer obvious explanations to the observed differences. Therefore, further investigations including data on the actual use of PSA testing, other influences (e.g., dietary and ethnic factors), and better data quality are needed to explain differences between the regions.

## Data Availability Statement

The original contributions presented in the study are included in the article/**Supplementary Material**. Further inquiries can be directed to the corresponding author.

## Author Contributions

AW and SK conceived and designed the study. SK, ES, CV, and JK acquired the data. SK and AW performed the literature search. SK performed the data analysis and ES supported. SK interpreted the data and ES, CV, and JK supported. SK performed the data management and wrote the manuscript. AW, GHB, ES, CV, and JK provided critical revision of the manuscript for important intellectual content. All authors contributed to the article and approved the submitted version.

## Funding

This study was funded by the Research Pool of Carl von Ossietzky University Oldenburg, Oldenburg, Germany (grant number: FP 2014-II_Winter).

## Conflict of Interest

The authors declare that the research was conducted in the absence of any commercial or financial relationships that could be construed as a potential conflict of interest.

## Supplementary Material

Supplementary Figure 1 Percentages of tumor (T) stage-specific prostate cancer cases for men aged 50 years and older in Lower Saxony and Groningen province from 2003─2012.

## Abbreviations

AAPC, Average annual percentage changes; APC, Annual percentage changes; ASIR, Age-standardized prostate cancer incidence rate; ASMR, Age-standardized prostate cancer mortality rate; ASR, Age-standardized rate; PLCO, Prostate, Lung, Colorectal and Ovarian Cancer Screening Trial; PSA, Prostate-specific antigen.
