# Supplementary material for: Differences in Prostate Cancer Incidence and Mortality in Lower Saxony (Germany) and Groningen Province (Netherlands): Potential Impact of Prostate-Specific Antigen Testing
Source: Front Oncol. 2021 May 28;11:681006. doi: 10.3389/fonc.2021.681006 (PMC8194402; doi:10.3389/fonc.2021.681006)
Supplement: Supplementary Figure 1 — Percentages of tumor (T) stage-specific prostate cancer cases for men aged 50 years and older in Lower Saxony and Groningen province from 2003─2012. [file DataSheet_1.docx]

**Supplementary Figure**


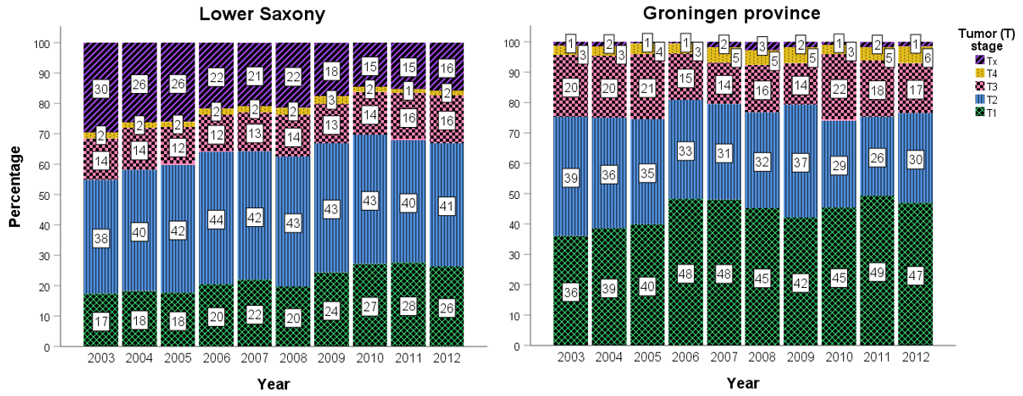


**Figure S1: Percentages of tumor (T) stage-specific prostate cancer cases for men aged 50 years and older in Lower Saxony and Groningen province from 2003─2012.**
